# A hierarchical approach for finding undiscovered populations of an endangered bumble bee

**DOI:** 10.1038/s41598-026-46861-8

**Published:** 2026-04-29

**Authors:** Clint R.V. Otto, Alma Schrage, Audrey Lothspeich, Larissa L. Bailey, Tamara A. Smith, Robert Planman, Judy Cardin, Kristen S. Ellis, Bethany Dennis, Ralph Grundel

**Affiliations:** 1 US Geological Survey, Northern Prairie Wildlife Research Center, 8711 37th St SE, Jamestown, ND USA; 2 US Geological Survey, Great Lakes Science Center, 1574 N 300 E, Chesterton, IN USA; 3 Department of Fish, Wildlife, and Conservation Biology, Colorado State University, Fort Collins, CO USA; 4 US Fish and Wildlife Service, Minnesota – Wisconsin Ecological Services Field Office, 3815 American Boulevard East, Bloomington, MN 55425 USA; 5 3009 Nottingham Way, Madison, WI 53713 USA

**Keywords:** Occupancy, Detection, Rare species, Pollinator, Presence-absence, *Bombus affinis*, Ecology, Ecology, Zoology

## Abstract

Understanding the distributions of rare species is necessary to guide monitoring and inform species recovery efforts. The rusty patched bumble bee (RPBB; *Bombus affinis,* Cresson) is an endangered species with an extant, known distribution centered around urban areas of the Midwestern United States. We tested a novel approach for finding undocumented RPBBs outside of urban centers and estimated the species occurrence at two scales that are relevant to management. We confirmed presence of RPBBs at 54% of the sampled 100 km^2^ grid cells where the species was previously undocumented, expanding the species’ known distribution by 5700 km^2^. After accounting for imperfect detection, our occupancy model estimated the number of occupied grid cells was 67 of 105 sampled grids, suggesting our methods were effective for finding undiscovered RPBB sites. Occupancy within 100 km^2^ grids was positively related to the number of occupied neighboring units but was not related to the area of developed land within 100km^2^ grid cells or smaller subunits (i.e. 3.14 ha patches or roadside transects). We highlight the utility of our approach for guiding future survey efforts by identifying an additional 145 grid cells where the occupancy status of RPBB is unknown but we predict a relatively high likelihood of RPBB occurrence. Our approach can be extended to find undiscovered RPBB sites in other areas and applied to other bee species where occurrence information is lacking outside of their core distribution.

**Supplementary Information:**

The online version contains supplementary material available at 10.1038/s41598-026-46861-8.

## Introduction

Understanding why some species are rare and other species are common is a foundational topic in ecology, especially in the context of biodiversity conservation^1^. Species rarity is a multidimensional trait that includes measures of local abundance, habitat specificity, and the extent of a species’ geographic distribution^2,3^. For example, some species exhibit high local abundances but are rare across much of their range, such that only a small fraction of spatial sampling units would be occupied^4^. Rarity increases the challenges associated with detecting species’ declines, particularly when estimating abundance. Rare species may be difficult to detect such that variability in count data may not reflect actual changes in population size or proportion of sampling units occupied by the target species^4^. Community science efforts, biological inventories, atlases, and biodiversity databases have made it easier for conservation biologists to understand patterns in species rarity and diversity, but even these rich data sources can be spatially and temporally biased^5–7^. For example, community science efforts are often heavily weighted towards urban systems, where increased sampling effort can inflate local abundance estimates and mask trends occurring across a species’ range^8,9^. Understanding the spatial distribution of rare and at-risk bumble bees is a primary goal of many recent inventory and monitoring projects in the United States^10,11^.

The rusty patched bumble bee (RPBB; *Bombus affinis,* Cresson) is a federally endangered species in the United States (U.S.) that was once widespread across the Midwest and Eastern U.S. and into Canada; its pre-1990 range has been reduced by ~ 90%^12,13^. Although the causes of RPBB declines are not definitively known, pathogens may have played a role^12^. The U.S. Fish and Wildlife Service (USFWS) tracks occupancy trends across a network of 100km^2^ grid cells to evaluate recovery progress across the historical distribution of RPBBs. Establishing contemporary information on the current distribution of RPBBs is an important first step in monitoring long-term population trends^11^. The USFWS drafted a long-term recovery plan that outlines several recovery criteria and developed monitoring guidance for tracking progress towards recovery goals^14^. Recent occurrence records of RPBB are concentrated near urban centers in the Upper Midwest^14^, and the distribution of RPBBs appears to be positively associated with human-developed lands^15,16^. In 2024, the USFWS proposed to designate 1.6 million acres of Critical Habitat for RPBB, within an area largely consisting of Midwestern cities^17^. However, researchers have pointed out that most recent occurrence records of RPBB that are centered around cities may be an artifact of non-random survey efforts in locations easily accessible by community scientists^15^. Although the number of RPBB detections has rapidly increased since 2017, thanks to community science data collection in urban areas from platforms such as iNaturalist, the Wisconsin Bumble Brigade, and Bumble Bee Watch, the overall occupancy trend of RPBB in the Midwest is static or decreasing^15^. Static occupancy trends over the known distribution may be due in part to limited survey effort outside of urban areas where RPBBs were found prior to their decline, and researchers have stressed the need for additional sampling in rural areas^15^. For example, RPBB detection data from iNaturalist highlights frequent observations of RPBB in and around cities of the Midwest (Available from https://www.inaturalist.org. Accessed 08.01.2023), but it is unclear if this is an artifact of substantial sampling effort or an affinity of RPBB to urban systems.

Limitations in current RPBB monitoring make it difficult to track species’ recovery goals and largely restrict monitoring inference to urban systems, where RPBB data collection is concentrated. The need for formalized monitoring programs for native bees has been identified as a national priority in the United States to understand population changes, inform status assessments, and evaluate recovery^10,18,19^. Understanding where an at-risk species such as the RPBB does and does not occur is an important first step when developing biological monitoring efforts^10^. Our objectives were to test a hierarchical approach for finding undiscovered RPBB locations and to estimate RPBB occupancy at two spatial scales while accounting for expected variation in detection probability across the sampling season. Specifically, we estimated occupancy at: (1) 100km^2^ grid cells (i.e. primary units), corresponding to the scale at which the USFWS is tracking RPBB recovery and (2) four, 3.14 ha patches and roadside transects (i.e. subunits) located within a grid cell, corresponding to the scale where most bumble bee surveys are conducted. Because our study was conducted in areas where occupancy status was unknown, we wanted to determine if RPBB occupancy exhibited positive correlations with the area of developed (i.e. urban) lands^15,16^ and if detection probabilities varied across the sampling season as seen in other studies^20,21^. We also tested if 100 km^2^ grid cell occupancy of RPBBs was positively related to the number of neighboring (i.e. sharing a side or vertex with) grid cells that were occupied by RPBBs. Finally, we apply the results of our analysis to predict additional grid cells throughout the Midwest that have a chance of being occupied by RPBB, but lack survey data.

## Results

We selected 569 potential subunits (i.e. 3.14 ha patches or roadside transects), across 131 100 km^2^ grid cells (i.e. primary units), for initial reconnaissance. Our field teams determined that 363 of the selected subunits had evidence of current and future floral resources and were accessible for RPBB surveys; the other 206 potential subunits had insufficient floral resources or accessibility issues. In some cases, an entire grid cell was removed from our sampling pool due to insufficient habitat or site access. Our field teams identified 84 additional subunits during their initial field reconnaissance trips that were added to our sampling pool. In several cases, field teams did not conduct surveys at all four subunits within a 100 km^2^ grid cell because RPBBs were already detected within one of the subunits and our protocols ended all surveys within a 100 km^2^ grid cell once RPBBs were detected. In these cases, none of the remaining subunits within the 100 km^2^ grid cell were surveyed and these unsurveyed subunits were excluded from the analysis. Our final sampling design consisted of 105 100 km^2^ grid cells, 301 subunits, and 1294, 30 minute surveys. Sampled grid cells had an average of 3.4 (+ /− 1.9, 1SD) neighboring grid cells with confirmed occurrences of RPBBs (hereafter “occupied neighboring grid cells”) and 0.22 (+ /− 0.24, 1SD) proportion of developed land within each grid cell. Subunits had 0.21 (+ /− 0.24, 1SD) proportion of developed land within a 500 m buffer of the subunit centroid.

**Fig. 1:**
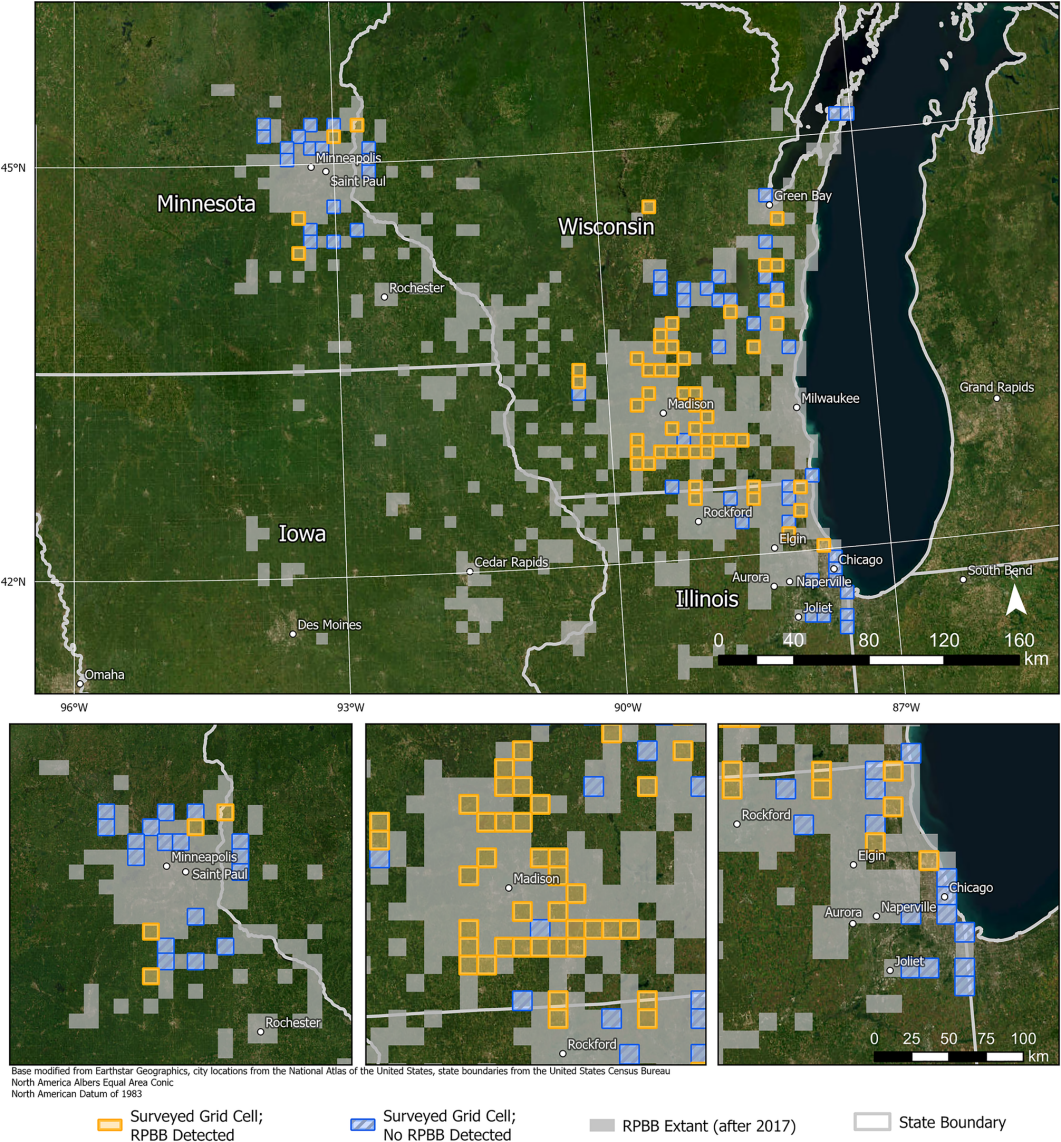
Known occupancy of the endangered Rusty Patched Bumble Bee (RBPP, *Bombus affinis*) across 100 km^2^ grid cells in the Midwestern United States. Grey cells represent cells with known occupancy, prior to our study. Blue and gold cells represent cells where RPBB occupancy status was unknown that were selected for bumble bee surveys. Gold cells are ones where our team detected RPBBs and blue cells are ones where our team surveyed but did not detect RBBBs. All map panels were generated with ArcGIS Pro 3.5.5 using a modified version of the world imagery basemap provided by Esri (Copyright Information—ArcGIS Pro | Documentation).

**Fig. 2:**
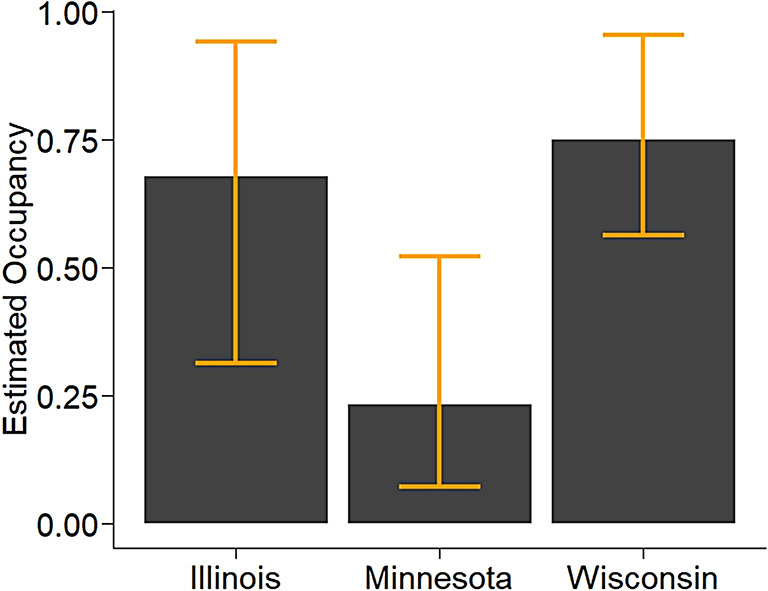
Estimates of the Rusty Patched Bumble Bee (RPBB, *Bombus affinis*) occupancy across 100km^2^ grid cells in Illinois, Minnesota, and Wisconsin from 2022–2024. Grid cells included in this study had no positive detections of RPBB since 2017. Orange bars represent 95% credible intervals.

**Fig. 3:**
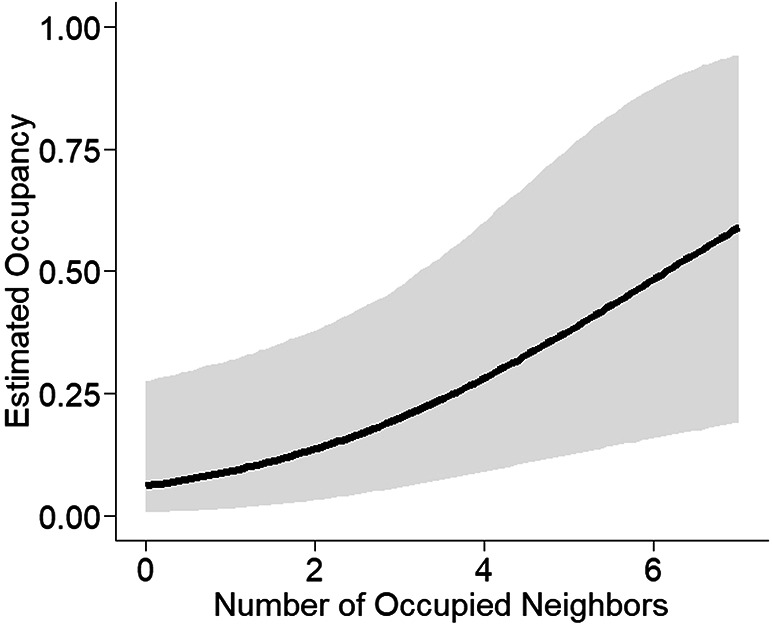
Relationship between Rusty Patched Bumble Bee (RPBB, *Bombus affinis*) occupancy probabilities across 100 km^2^ grid cells in Illinois, Minnesota, and Wisconsin from 2022 to 2024 and the number of neighboring grid cells that were occupied by RPBBs. Grey shaded region represents the 95% credible interval. Grid cells included in this study had no positive detections of RPBB since 2017.

We detected RPBB at 57 of 105 sampled grid cells, expanding the known distribution of RPBB in the Midwest by 5700 km^2^ (Fig. 1). After accounting for imperfect detection, the estimated number of occupied grid cells was 67 (95% Credible Interval: 60–79), suggesting that occupancy of grid cells that met our selection criteria was relatively high. However, occupancy estimates varied across the three states (Fig. 2). Grid cell occupancy in Wisconsin was highest among the three states, with an estimated 0.75 of the grid cells being occupied by RPBBs, whereas occupancy in Minnesota was ≈0.25. Illinois occupancy estimates were in between Wisconsin and Minnesota with an estimated 0.67 of sampled grid cells occupied by RPBBs. There was a positive relationship between RPBB grid cell occupancy and the number of known occupied neighboring grid cells (Fig. 3). For example, expected occupancy for grid cells with 6 known occupied neighboring grid cells was 79% higher than grid cells with only 4 known occupied neighbors. Counter to our initial prediction, RPBB occupancy was not positively related to the area of developed land at the grid cell or the subunit scale (Fig. S2). There was evidence of a negative relationship between RPBB occupancy and the area of developed land within grid cells; however, the 95% credible intervals overlapped zero (Fig. S2A). The average probability that RPBB occurred at a subunit, given the grid cell was occupied, was 0.58 (95% CI 0.37–0.80) and not strongly influenced by the amount of developed land in the 500-m buffer (Fig. S2B).

**Fig. 4:**
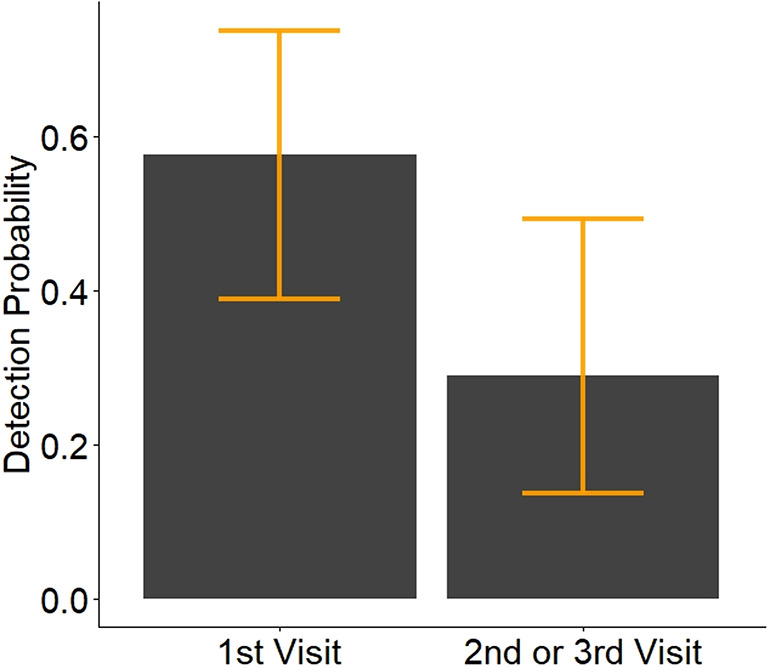
Estimated probability of detecting Rusty Patched Bumble Bee (RPBB, *Bombus affinis*) during a 30 min survey conducted by a single observer, given the 3.14 ha subunit unit was occupied. Separate detection probabilities were estimated for surveys occurring during the mean day of year (218, ≈ 06 August) for the first visit (surveys 1 and 2), and second and third visit (surveys 3 through 6) to a subunit. A maximum of 6 surveys were conducted at each subunit and surveys were terminated within all subunits once RPPB was detected with the 100 km^2^ grid cell. Orange bars represent 95% credible intervals.

Detection probability for RPBB was higher during the first visit to an occupied subunit, suggesting that RPBBs were likely to be detected during the initial visit, relative to the second or third visit (Fig. 4). Of the 61 positive RPBB detections, 44 were made during the first visit (Table S1). Observers were most likely to detect RPBBs from August into early September (day of year 215–250, Fig. S3). Estimates of RPBB detection probability were relatively high during the last third of our survey period (Fig. S3); however, parameter estimates were also imprecise during this period, likely due to the relatively few surveys our team performed during this time period. Several positive detections of RPBB in late August into September in 2022 at newly surveyed subunits were likely responsible for the high detection estimates during the latter portion of the sampling period.

**Fig. 5:**
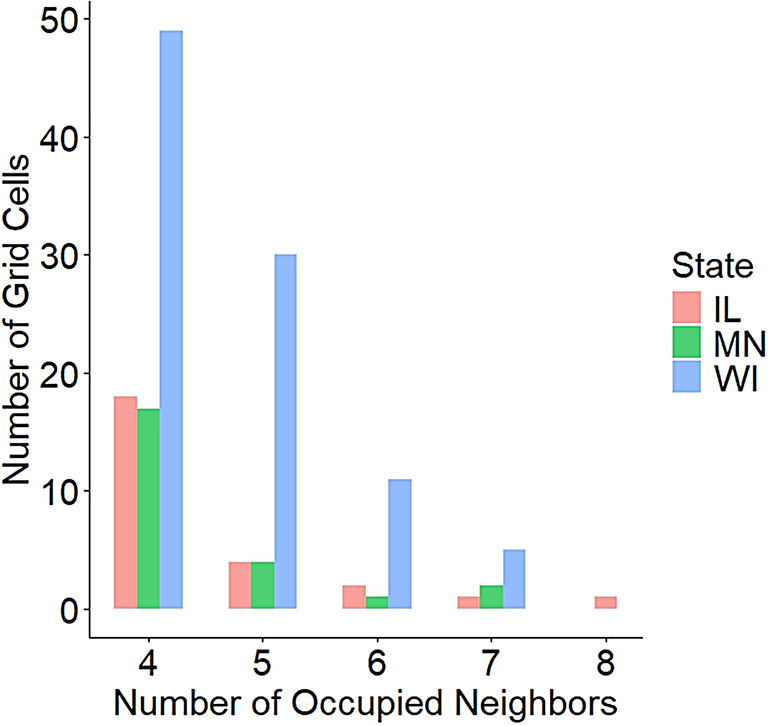
Number of 100 km^2^ grid cells that did not have prior record of Rusty Patched Bumble Bee (RPBB, *Bombus affinis*) occurrence and were not surveyed as part of the current study. Based on model results (Fig. 4) these grid cells have a likelihood of RPBB occupancy and may be considered priority grids for future surveys, to better quantify the known distribution of RPBBs. X-axis is the number of neighboring 100 km^2^ grid cells that are occupied by RPBBs, color coded by state (IL = Illinois, MN = Minnesota, WI = Wisconsin).

Our work can easily be extended to prioritize sampling of additional grid cells with a high number of occupied neighboring grid cells but with no recorded survey effort in recent years (Fig. 5). Specifically, there are 145 grid cells across our three-state region with no records of RPBB since 2017 and that also have ≥ 4 occupied neighboring grid cells (Fig. 5). Occupancy estimates in our study suggest ~ 30–60% of these grid cells are likely occupied by RPBB but have limited or no reported survey effort.

## Discussion

We tested a hierarchical approach for finding undiscovered RPBB sites and estimated occupancy at two spatial scales across the Midwest. To our knowledge, this is the first completed study to conduct multi-state sampling in areas with no prior knowledge of RPBB occurrence since the species was listed under the Endangered Species Act (ESA). Model estimates revealed a majority (67, 95% credible interval: 60–78) of the 105, 100 km^2^ grid cells we surveyed were likely occupied by RPBB. Visit-specific estimates of RPBB detection probability showed the best chance of detecting RPBB was during the first visit to a subunit (Fig. 4), further demonstrating the effectiveness of this approach for efficiently finding undiscovered RPBB sites. This study holds promise for informing RPBB recovery efforts, suggesting that additional RPBB sites await discovery. Our research can be used to help monitoring programs prioritize future sampling efforts by identifying sampling units where the status of RPBB is currently unknown, but the likelihood of RPBB occurrence is relatively high. This form of systematic sampling, with multiple repeated surveys, may eventually lead to a more accurate projection of the distribution of RPBB outside of urban areas, where inventory and monitoring efforts are lacking. Furthermore, our research can guide sampling unit selection for nascent inventory and monitoring efforts when finding suitable locations for monitoring is challenging. Spatially unbiased monitoring would be beneficial not only for RPBBs^14^ but also for many of the + 4000 native bee species in the United States that are data deficient^10^. Collectively, these results demonstrate the utility of our approach for finding undiscovered populations of RPBBs and provide estimates of occupancy at two spatial scales that are relevant to managers.

Occupancy estimates were substantially lower in Minnesota, relative to Wisconsin and Illinois. Determining the causes of state differences in occupancy is beyond the scope of this study, but we offer four potential hypotheses for future exploration. First, variability in the depth and breadth of previous monitoring efforts in each state may be responsible for the observed differences in occupancy. Our grid cell selection was determined by information from the USFWS Priority Grid Map^34^, which in turn is informed by the data collected by hundreds of individual researchers and monitoring efforts nationwide. Minnesota has a longer history of coordinated bumble bee monitoring efforts compared to Wisconsin and Illinois, although in recent years the latter two states have expanded bumble bee community science projects. It is possible that in Minnesota most of the occupied grid cells have already been discovered through past surveys, leaving fewer undiscovered, occupied grids cells for our team to survey. Alternatively, severe drought conditions in Minnesota in 2022 and 2023 may have been responsible for lower occupancy estimates in Minnesota, although drought conditions were widespread through all states and years sampled (Historical Data and Conditions | Drought.gov https://www.drought.gov/historical-information?dataset=0&selectedDateUSDM=20260324). Thirdly, unmodeled differences in habitat quality may explain variation in occupancy. Although we did not detect a relationship between RPBB occupancy and developed land, it is possible that other unmodeled factors such as proximity to pesticide exposure, floral resource quality, or other factors that we were unable to measure were responsible for patterns in occupancy. Finally, it is possible that our occupancy estimates are reflective of differing population trends of RPBBs where populations in Minnesota may be stable or declining and populations in southern Wisconsin and Illinois may be expanding. Ultimately, our research emphasizes the need for long-term monitoring to understand temporal and spatial patterns, and environmental drivers of those patterns, of RPBB occupancy^22^.

Counter to our initial prediction, occupancy at neither the 100 km^2^ grid cell nor subunit (i.e. 3.14 ha patches or roadside transects) scale was positively associated with the area of developed lands. In fact, our analysis suggested a negative association between RPBB occupancy and developed land area within 100 km^2^ grid cells; however, the 95% credible intervals overlapped zero, indicating considerable uncertainty in the estimate. These findings are seemingly in conflict with recent research showing RPBB occupancy is positively related to developed land^15,16^. This difference may be due to differences in our sampling unit selection procedures and inferential space, compared with previous studies. Many of the sampling units selected in Boone et al.^16^ with a high proportion of developed land were within the metropolitan region of Minneapolis and St. Paul, Minnesota, a known stronghold of RPBBs. However, no additional urban areas were included in the study by Boone et al.^16^, which limits the inferential space over which the urban association can be applied. The analysis conducted by Ellis et al.^15^ was based largely on community science data and therefore weighted towards urban systems. Our sampling was conducted outside of any known RPBB stronghold or urban areas that have been heavily surveyed by community scientists. Collectively, Boone et al.^16^, Ellis et al.^15^, and our current study suggest that urban centers are important strongholds for RPBBs but that the species is not confined exclusively to developed areas. This finding is supported by other research conducted outside of the Midwest that showed RPBBs are not confined exclusively to urban systems^23^. Alternatively, the urban association observed by Boone et al.^16^ and Ellis et al.^15^ may represent an ecological phenomenon where urban environments provide refugia against potential threats such as pathogens and pesticides and therefore support higher rates of occupancy, yet our study failed to detect this important association due to limited sampling in high density urban environments.

It is important to note that findings from our study do not contradict the USFWS’s proposed Critical Habitat designation that encompasses major metropolitan areas of the upper Midwest^17^. “Critical Habitat” is a legally designated geographic area essential to the conservation of a U.S. federally listed species^24^. Urban areas proposed as Critical Habitat in the draft USFWS document^17^ are likely very important for RPBBs. Our study shows that areas outside of these urban centers also support extant populations of RPBBs. Future surveys could determine how widespread the species is and how stable these newly discovered populations are, outside of urban systems. It is unclear if the populations we discovered are resistant and resilient to changes in occupancy state through time, an issue that could be addressed through long-term monitoring. Understanding the underlying processes that drive changes in species occupancy through time (i.e. colonization and extirpation) is an integral yet often overlooked component of wild species monitoring^22^. Our study shows that RPBBs are less restricted to urban areas than would be expected based on recent distributional information^15,16^.

One important limitation of our study is that we employed a removal design^25^ where a selected 100km^2^ grid cell was removed from the sampling pool once RPBB was detected at one or more subunits within the grid cell. This removal design was adopted to maximize our ability to find undiscovered populations of RPBBs at the 100km^2^ grid scale, but also limited our ability to estimate occupancy across subunits. For example, RPBBs were detected within 47 100km^2^ grid cells during the first visit to the subunit (i.e. first two 30-min surveys). These 100km^2^ grid cells were then removed from the sampling pool with no further surveys conducted at the other subunits within the grid cell. Although this approach was effective at finding new populations of RPBBs within 100km^2^ grids, it also limited our ability to generate reliable estimates of occupancy across subunits. We also note that our seemingly high estimates of occupancy within subunits (mean = 0.58) are limited to patches that meet our selection criteria (i.e. see Methods) and should not be applied to areas that do not meet these criteria. Thus, small-scale occupancy of RPBBs is likely much lower in areas that do not meet our sampling unit selection criteria. Additional research could help obtain more robust estimates of RPBB occupancy across secondary subunits (i.e. small-scale, patch level) and infer land cover and habitat associations at this smaller scale. Similarly, our removal design was likely responsible for the decreased precision of detection estimates over the season (Fig. S3) because sampling at many of the subunits was terminated before completing six surveys. This finding is consistent with Mackenzie and Royle^25^, who found that removal designs are less flexible in exploring potential sources of variation in detection probability than are standard designs, in which each sampling unit receives the full complement of replicate surveys.

Formalized monitoring efforts of rare species often include a proportion of sampling units where the occupancy status of the focal species is previously known, and sampling units where the occupancy status is previously unknown but the species has some probability of occurrence. Sampling across these two site types across time may eventually allow for estimation of occupancy dynamics, specifically the likelihood of known occupied units becoming unoccupied (i.e. local extirpation) and the likelihood of currently unoccupied units becoming occupied in the future (i.e. local colonization,^26^). Monitoring in areas where occupancy of the focal species is currently unknown also provides a more accurate depiction of the species distribution, as opposed to one largely based on community science records centered around urban areas (e.g.^15^). One of the premier challenges for monitoring rare species, including bees, is having a spatially unbiased sampling approach, where a portion of sampling locations are selected for long-term monitoring without prior knowledge of species occupancy^22,27^. If all sampling units are selected only from locations where the species is known to occur (i.e. selecting occupied sites), occupancy trend estimates will only be applicable to other units where occupancy status is known and not representative of a larger group of units where occupancy status is unknown^27^. Addressing this known limitation in bee monitoring requires selection of a subset of units where the current occupancy status is unknown, but the species has reasonable probability of occurrence^22,28^. In this light, our sampling unit selection process can be used to probabilistically incorporate sampling units into formal monitoring efforts where occupancy status of focal species is unknown, but there is a reasonable chance of occupancy. For example, our study has identified 145, 100km^2^ grid cells that have a reasonable likelihood of RPBB occupancy but little to no survey effort has been reported in our three-state region (Fig. 5). These grid cells represent a population of units that could be included in long-term monitoring where occupancy dynamics could be readily estimated.

Our approach can easily be extended to find undiscovered populations of RPBBs in other regions and extended to other bee species for which baseline distribution information is available but considerable sampling gaps in their known range exist. Specifically, there is an opportunity to apply our approach to areas that represent the current, peripheral distribution of RPBBs such as northwestern Indiana, western Upper Peninsula of Michigan, eastern Iowa, and portions of the Appalachian Mountains. In these areas, unknown grid cells could be prioritized based on the number of occupied neighboring grids, accessible public lands, land covers that support bumble bees, and community science records of flowers that are visited by RPBB. In addition, our approach can be applied to other bee species of conservation concern, particularly for species with spatially limited monitoring or data deficiencies that limit understanding of species’ current status and distribution. For example, our approach could be applied to multiple bumble bee species that are being considered for listing under the ESA or are listed as data deficient on State Wildlife Action Plans^29^. This assessment would expand the distribution data necessary to develop long-term monitoring efforts for estimating demographic trends through time, species response to environmental stressors, and the effects of management^30^. However, an important prerequisite for applying our methods is the existence of baseline distribution information. The rapid growth in bee inventory and atlas projects in recent years means these data are, or may soon be, available for a growing number of species. Other types of bee survey methods such as passive sampling devices, eDNA, and lethal capture can easily be incorporated into the approach we implemented. Finally, our study highlights the importance of accounting for imperfect detection when modeling bumble bee distributions, particularly when sampling in areas where the occupancy status is unknown. Understanding bee status and trends will inform the conservation status of bumble bees in the United States and internationally and possibly contribute to more informed Endangered Species Act listing decisions and subsequent recovery activities^11,31^.

## Methods

### Study area and study species

The historical distribution of the RPBB encompassed 29 states in the Eastern and Central United States and three Canadian provinces; however, the current (2017–2024), known distribution is confined to seven Midwestern and Mid-Atlantic states. The species is found in a wide variety of habitats such as fallow fields, roadsides, gardens, natural areas, and urban centers^32,33^, but the extant core distribution of the species in the Midwest appears to be currently largely confined to urban areas^15,16^.

Our study was conducted from 2022 to 2024 in Minnesota, Wisconsin, and Illinois, three U.S. states that encompass the majority of RPBB detections since the species was listed in 2017 and represent 75% of the states within RPBB Conservation Units 1 and 2^14^. Our sampling framework included three phases: (1) GIS-based selection of 100km^2^ grid cells and subunits, (2) field reconnaissance, and (3) RPBB visual encounter surveys. We conducted surveys in all three states in 2022 and 2023 but did not conduct surveys in Minnesota in 2024 due to lack of new RPBB detections in the previous years.

### *GIS-based selection of 100km*^*2*^* grid cells and subunits*

Our study was designed to estimate RPBB occupancy at two spatial scales: (1) 100km^2^ grid cells and (2) 3.14 ha patches or roadside subunits. Thus, we employed a hierarchical selection process to first select 100km^2^ grid cells that may support undiscovered RPBB populations. Within a given grid cell, there are numerous fields, meadows, roadside ditches, urban parks, wetland buffers, etc. that could serve as foraging habitat for RPBB. These patches of bumble bee foraging habitat represent potential subunits, or secondary units, that our field teams could survey for RPBBs.

**Fig. 6:**
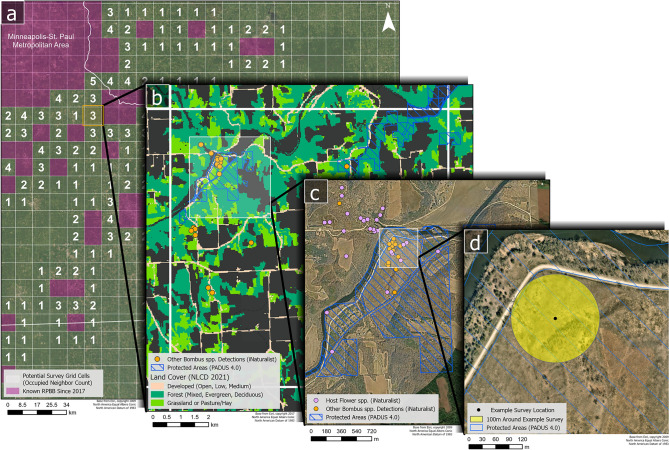
GIS selection process for locating subunits (i.e. 3.14 ha patch or roadside transect) to survey for undiscovered Rusty Patched Bumble Bee (RBPP, *Bombus affinis*) populations. All map panels were generated with ArcGIS Pro 3.5.5 using a modified version of the world imagery basemap provided by Esri (Copyright Information—ArcGIS Pro | Documentation). (**A**) Purple 100km^2^ grid cells are areas where the presence of RPBB had previously been confirmed by the USFWS. The number within each blank grid cell represents the number of adjacent, occupied neighboring grid cells. (**B**) An example grid cell where occupancy status is unknown, with 3 neighboring, occupied grid cells. Our team mapped land cover, public areas, other bumble bee detections and host plant detections to identify potential areas within a grid cell to survey for RPBBs. (**C**) Zooming into a specific area within a grid cell with past bumble bee detections and host plant detections (data from iNaturalist). (**D**) Example starting location for a 30-min RPBB survey within a 3.14 ha area. Our sampling design goal was to select 4–6 of these subunits per grid cell for initial reconnaissance and subsequent RPBB surveys if the subunit had sufficient floral resources.

We selected 100 km^2^ grid cells and subunits prior to each field season and repeated the process as needed during a field season if our field teams requested additional units. We used USFWS’s Priority Survey Grid Map^34^ to select 100 km^2^ grid cells where RPBB had not been detected since 2017 but had neighboring (adjacent) grid cells where RPBBs had been detected at least once since 2017 (Fig. 6A). We expected that grid cells with a high number of known occupied neighboring cells were more likely to be occupied by RPBBs than those with few or no occupied neighbors (each cell had 8 neighboring cells; Fig. 6A). There were no unknown grid cells with 8 occupied neighbors. Therefore, we prioritized selection of grid cells with 6–7 known occupied neighbors and a subset of cells with < 6 occupied neighbors within a reasonable driving distance once we surveyed all grid cells with 6–7 occupied neighbors (Table 1). We prioritized grid cells with < 6 occupied neighbors by availability of public land, bee-friendly land cover, evidence of floral resources, and suitable driving distance for our field teams (explained below, Fig. 6). We ensured we had representative grid cells with 1–7 occupied neighbors to allow us to estimate the relationship between RPBB occupancy and the number of occupied neighbors (Table 1). Due to funding requirements in 2023, we sampled five grid cells in Illinois that were in the Great Lakes watershed but had zero occupied neighbors (Table 1). When a grid cell was selected for sampling, all bumble bee surveys were performed within the current calendar year (i.e. cells were not surveyed over multiple years).

**Table 1:** Number of 100km^2^ grid cells selected for occupancy assessment, 2022–2024.

Occupied neighbors	Illinois	Minnesota	Wisconsin
0	5	0	0
1	2	0	13
2	1	7	5
3	2	2	21
4	3	3	10
5	5	1	9
6	1	3	4
7	2	1	5
8	0	0	0

Occupied neighbors are the number of adjacent, 100 km^2^ grid cells (i.e. sharing a side or vertex) where *Bombus affinis* had been detected at least once since 2017. Each grid cell shares a side or vertex with eight other grid cells.

Within each selected grid cell, our goal was to survey four subunits for previously undiscovered RPBB populations. Ideally, a subunit would consist of a 100-m radius plot of foraging habitat (3.14 ha circular plot); however, in grid cells with little public land (explained below) we also surveyed roadside habitat, where we defined the subunit as 150 m in either direction from a starting location and 50 m from the road shoulder into the verge. The size of each subunit was based on a previous study where 3 ha was identified as a reasonable area to cover during a 30-min survey^21^.

In selected grid cells, we identified potential subunits by identifying areas (1) with land covers that support foraging RPBB, (2) were publicly owned, (3) had Research-Grade iNaturalist observations of other *Bombus* (bumble bee) species dating from 2014 up to spring of the year of survey^35^, *Bombus* observations from Wisconsin Bumble Bee Brigade up to spring of 2023 (Wisconsin Bumble Bee Brigade), and Research Grade iNaturalist records^36^ of 37 known RPBB floral hosts^21,37^, and (4) were within a reasonable driving distance for field teams (Fig. 6B,C). Dominant land cover types supporting RPBB foraging include deciduous forest, herbaceous grassland, and lower intensity developed spaces (refer to Supplementary Information, Fig. S1). We used the USGS Protected Areas Database^38^ to identify public lands with our targeted land cover types with easy access from roads or trails (Fig. 6B). We focused on public lands to align our study with community science monitoring programs such as the Wisconsin Bumble Bee Brigade and Minnesota Bumble Bee Atlas. Our assumption was that if community scientists had recently detected RPBB forage plants or other bumble bee species at a specific location, that location represented potential foraging habitat for RPBBs. This step allowed us to take a fine-filter approach to selecting subunits where RPBBs had not been detected but bumble bee habitat was likely to occur. Through this process we assembled GIS layers that identify public lands where the dominant vegetation type was herbaceous grassland, or some form of lower-intensity developed land, and where RPBB host plants and other bumble bees have been detected (Fig. 6C). We then used aerial imagery to hand-select 4–6 points, corresponding to the centroid of potential subunit locations, in each grid cell for field reconnaissance (Fig. 6D).

### Field reconnaissance

Prior to conducting RPBB surveys, we visited the potential subunits within a selected grid cell to determine whether subunits were accessible to field teams and whether they possessed sufficient floral resources for foraging bumble bees, either during the visit or (likely) during future visits. Field teams were not required to use the centroid point identified in the GIS unit selection – typically in the act of navigating to a point, the team could determine an appropriate place to center a subunit based on the distribution of flowers currently blooming or due to bloom in the near future. Once the center point was determined, it was fixed for all surveys of the subunit. All subunits within a grid cell were separated by > 1 km from other suitable subunits.

Field teams conducted reconnaissance in June prior to the survey season in all years, but in 2023 and 2024 teams also conducted reconnaissance on a rolling basis during the field season when the weather or logistical constraints limited bumble bee surveys (e.g. rain or cold mornings). We recorded the number of potential subunit points selected via GIS that were removed during reconnaissance due to insufficient floral resources or due to access or safety issues.

### Visual encounter surveys

Previous research showed six, 30-min visual encounter surveys are required to achieve a high likelihood of detecting RPBBs at a ~ 3 ha sampling unit^21^. Thus, our goal was to conduct a maximum of six surveys for RPBBs at each subunit, with 4 subunits within each grid cell (i.e. 24 potential surveys within a grid). We employed a ‘removal design’ such that once RPBBs were detected at a subunit within a 100km^2^ grid cell, we immediately ceased surveys at all subunits in that cell and moved on to another grid cell. While this approach likely reduced our ability to obtain precise estimates of RPBB detection probability and occupancy at the subunit scale, it allowed the field teams to move quickly to new grid cells during the field season and conduct more surveys in grid cells with unknown RPBB occupancy status.

Each subunit was visited up to three times during the field season (i.e. time period between visits explained below), with each visit consisting of two surveys. A survey consisted of a single observer conducting a timed, 30-min random walk, observing bumble bees within 100 m of the centroid location for circular subunits and within 150 m of starting location for roadside surveys. Two independent observers surveyed the subunit simultaneously but were instructed to stay separated and not share detection information. Observers were instructed to scan the subunit for bumble bee activity and move towards those areas. If bumble bees were not obvious, observers were instructed to travel from flower patch to flower patch looking for active bumble bees. Observers documented detections of RPBBs by collecting photographic vouchers that were later verified by coauthor Alma Schrage. Photos were required to confirm positive detections, and field teams indicated obtaining photographic vouchers of RPBBs was relatively easy. Observers also recorded flowering forbs visited by RPBBs. For surveys where just a single observer was available, the observer completed two consecutive 30-min surveys. If a RPBB was detected during the first 30-min survey the single observer did not complete a second survey. Table S1 provides a summary of the number of surveys conducted by our field teams.

We initiated surveys on June 17 in 2022, but we delayed until July 1 in 2023 and 2024 because of the lack of early-season detections in 2022. Our field teams completed six surveys at each subunit unless RPBB was detected at a subunit within the grid cell, whereupon teams terminated future surveys to all subunits within that grid cell. Field teams separated revisits to subunits by 3–10 days in 2022 and 10–17 days in 2023 and 2024, following a better understanding of floral resource flowering timing. Surveys ended by mid-September each year. Observers did not conduct surveys during rain, when wind speed was > 20kph (12mph) or temperatures were < 15.5 °C (60°F). Observers waited at least one hour after rain subsided before conducting a survey. Surveys on partially cloudy days or during overcast conditions were permissible if observers could see their shadow, or if bumble bees were observed foraging at the subunit. All surveys were conducted at least 2 h after sunrise and 2 h before sunset. Prior to each survey, observers recorded wind speed (kph), air temperature (°C), relative humidity (%), and estimated cloud cover (%) with a Kestrel ® Pocket Weather Station or weather app that provided location-specific weather information.

### Data analysis

We expected RPBB occupancy across 100km^2^ grid cells to be positively associated with the amount of developed land within each grid cell^15,16^. We determined the dominant landcover within 100km^2^ grid cells by tabulating the 2021 National Land Cover Database (NLCD). We defined a buffer around subunits as a 500-m radius around each subunit location, similar to Boone et al.^16^. We calculated the count of 30 m^2^ NLCD pixels by all land cover classes within the grid cell with the `rasterstats` package in Python^39^ and included only pixels with a centroid within the grid cell. We combined pixel counts for all land cover classes that represent development (‘Developed, Open Space’, ‘Developed, Low Intensity’, ‘Developed, Medium Intensity’, and ‘Developed, High Intensity’) and used this as our ‘*developed land’* covariate in our subsequent multi-scale occupancy analysis. This reclassification of developed land was also used by previous studies that showed a positive relationship between RPBB occupancy and area of developed land^15,16^. We also expected a positive association between the area of ‘developed land’ and subunit occupancy. To model this relationship, we followed the above procedure but quantified developed land within a 500 m radius of the subunit centroid.

We used a multi-scaled occupancy model^40^ to estimate RPBB occupancy at our two spatial scales, while accounting for species imperfect detection (refer to Supplemental Material for complete model code). This hierarchical model consists of three parameters:

$\psi$_i_ = the probability RPBB occurs in a 100 km^2^ grid cell *i* (where *i* = 1–105).

${\theta }_{i,j}$ = the conditional probability that RPBB occurs at subunit *j* (i.e. 3.14-ha circular plot or roadside subunit), given the species is present in the 100km^2^ grid cell, *i* (primary unit) (where *j* = 1, 2, 3, 4).

${p}_{i,j,k}$ = the conditional probability of detecting RPBB during survey *k* (i.e. 30-min Visual Encounter Survey) conducted in subunit *j* of grid *i*, given the species is present at the subunit, where *k* = 1–6).

This modeling approach allows for the estimation and modeling of occupancy at the scale of RPBB conservation grid cells ($\psi$) and subunits within an occupied grid cell (θ). The product of these two parameters, ${\widehat{\theta }}_{c}= \widehat{\uppsi }* \widehat{\uptheta }$, represents the conditional probability of “small scale” occupancy or the probability that a subunit (i.e. 3.14-ha circular plot or roadside subunit) is used by foraging RPBB^40,41^. This model uses detection history data (i.e. 1–6, 30-min surveys) collected at subunits within each sampled grid cell to estimate occupancy ($\psi$ and θ) and detection (*p*) probabilities.

We fit a single multi-scale occupancy model in a Bayesian framework: $\psi$ (state + neighbors + developed land), *θ* (developed land subunit), p(1st_visit + day of year + day of year^2^). *State*, *neighbors*, and *developed land* (within a grid cell) are grid-specific covariates that vary among the *i* grid cells:

$${\mathrm{logit}}(p_{i,j,k} ) \, = \beta_{5} + \beta_{6} *{\mathrm{1}^{st}}{visit}_{i,j,k } + \beta_{{7}} *{\text{day of year}}_{i,j,k} + \beta_{{8}}*{\text{day of year}^2}_{i,j,k}.$$

We estimated RPBB grid cell occupancy ($\psi$) and allowed it to vary as a function of *state* (Illinois, Minnesota, Wisconsin)*.* Although generating state-specific estimates was not a primary objective, reports from field teams indicated substantial differences in RPBB detections across states. Furthermore, natural resource managers are interested in having state-specific estimates of RPBB occupancy. We included the number of occupied *neighbors*, out of eight possible, as a covariate on $\psi$. We predicted occupancy within a grid cell would be higher for cells with more occupied neighboring grid cells. We estimated the number of occupied 100km^2^ grid cells as a derived parameter by summing the latent occupancy state variable (z_i_ ~ Bernoulli ($\psi$_i_)), which represents whether each grid cell was occupied or unoccupied^42^. Lastly, we included the area of *developed land* within each grid cell as a factor to explain variation in RPBB occupancy among grid cells.

We also expected subunit occupancy (θ) would be influenced by the amount of developed land around a subunit, quantified using a 500 m buffer around the subunit centroid. We termed this covariate “*subunit developed land*” as it is a subunit-specific covariate that varies among all *j* subunits, i.e. among all *j* subunits within each grid cell *i*:

logit(θ_*i,j*_) = $$\beta$$_3_ + $$\beta$$_4_ * subunit_developed_land_*i,j*_

For both *developed land* and *subunit developed land* covariates we predicted RPBB occupancy would be positively related to the area of developed land^15,16^.

We allowed detection probabilities (p) to vary as a function of *day of year*. We fit *day of year* as a quadratic function to account for known peak activity period and RPBB colony size^15^. We also include a *first visit* detection covariate where a separate detection probability was estimated for 1) 30-min surveys conducted during the initial visit to a subunit (*i.e.* surveys 1 and 2), and 2) surveys conducted during the second or third visit to the subunit (i.e. surveys 3—6). This model structure is analogous to animal mark-recapture studies that employ a removal design to estimate abundance, where researchers expect higher capture rates the first time a population is surveyed^43^. Likewise, we expected detection probability of RPBBs to be highest the first time a subunit was visited, relative to subsequent visits. Including the “*1st visit”* covariate allowed us to account for differences among occupied subunits that have (1) robust populations of RPBB in the vicinity, and thus we were more likely to detect the species during the first visit regardless of time of year, versus (2) smaller populations of RPBB where more sampling effort may be required, ideally during a time period when RPBB worker abundance is at its peak. *Day of year* and *1st visit* were covariates corresponding to the day of year when survey *k* was conducted and whether survey *k* was conducted during the first visit to the subunit, respectively:

logit(p_*i,j,k*_) = $\beta$_4_ + $\beta$_5_ * 1st_visit_*i,j,k*_ + $\beta$_6_ * day of year_*i,j,k*_ + $\beta$_7_ * day of year^2^_*i,j,k*_

We fit our occupancy model with JAGS^44^ in R 4.2.^45^ via the jagsUI package^46^. Model code is available in the Supplementary Material. We used large variance priors for all intercepts and covariate effects, and we present mean and 95% credible intervals to determine importance of covariates. All covariates were standardized to mean of zero and variance equal to one prior to analysis and then back-transformed during figure production. We used 3 chains of 25,000 iterations with 5000 iterations discarded during the initial burn-in period and thinned to yield 5000 samples. We assessed model convergence by examining the Gelman-Rubin statistic (< 1.1, Ref. 47).

## Supplementary Information

Supplementary Information.

## Data Availability

USGS-collected data for this study are available on the US Geological Survey ScienceBase Catalog^48^.
