# Supplementary material for: A hierarchical approach for finding undiscovered populations of an endangered bumble bee
Source: Sci Rep. 2026 Apr 29;16:13759. doi: 10.1038/s41598-026-46861-8 (PMC13128823; doi:10.1038/s41598-026-46861-8)
Supplement: Supplementary file 1 — Supplementary Information. [file 41598_2026_46861_MOESM1_ESM.docx]

Supplementary Information

Table S1. Distribution of the 1,294 30-minute rusty patched bumble bee (RPBB) surveys conducted across 3.14 ha patches or roadside transects (i.e. subunits) located within 100km^2^ grid cells in Illinois, Minnesota, and Wisconsin, 2022-2024. Surveys were most often paired (1-2, 3-4, 5-6) where two independent observers conducted surveys during the same visit to the subunit. For some surveys two observers were not available. In these cases, a single observer conducted a 30-minute survey and then conducted a second survey immediately after the first survey if RPBBs were not detected during the first survey, but did not conduct a second survey if RPBBs were detected during the first survey. “Number of Positive Detections” refers to the number of surveys where RPBB were detected. There were 4 occasions where RPBBs were detected in more than one subunit within a grid cell. This happened when field crews were unable to report a positive detection of RPBBs to other field teams until the end of the field day. In these rare cases, RPBBs were discovered in multiple subunits within a grid cell which is why there were 61 positive detections within subunits but only 57 grid cells with confirmed occupancy (see Results).

| Survey Replicate | Number of Surveys | Number of Positive Detections |
| --- | --- | --- |
| 1 (1st Visit) | 266 | 34 |
| 2 (1st Visit) | 256 | 10 |
| 3 (2nd Visit) | 204 | 5 |
| 4 (2nd Visit) | 198 | 7 |
| 5 (3rd Visit) | 156 | 3 |
| 6 (3rd Visit) | 153 | 2 |
| Total | 1294 | 61 |

JAGS Code for Multi-scaled Occupancy Model

Code to implement multiscale occupancy analysis (Nichols et al. 2008) which estimates grid cell occupancy (psi), subunit occupancy (theta), and detection probabilities (p) within a hierarchical framework. Data supplied to JAGS includes: 1) the detection history array (y) indexed by grid (*i*), subunit (*j*), and survey (*k*); 2) grid-level covariates (*state, neighbor, developed_land*) for the ecological state submodel indexed by grid (*i*); 3) a subunit-level covariate (*subunit_developed_land*) indexed by grid (*i*) and subunit (*j*); 4) survey-specific covariates for the detection submodel (*1^st^ visit, day of year*) indexed by grid (*i*), subunit (*j*), and survey occasion (*k*); 5) dimension and indexing constants defining the hierarchical structure (n.state, n.grid, n.unit, n.survey). Survey occasion, *k*, was indexed from 1-6, corresponding with two independent surveys conducted during three potential visits to each subunit. Surveys 1 and 2 correspond to Visit 1, surveys 3 and 4 to Visit 2, and Surveys 5 and 6 to Visit 3. These paired surveys share identical covariate values for *1^st^ visit* and *day of year* covariates but represent independent surveys for rusty patched bumble bees. Priors and derived parameters are provided in the model code.

model {

# Priors and model for parameters

# Intercept of occupancy probability

for(t in 1:n.state){

int.psi[t] ~ dnorm(0, 0.1) # Intercept of grid-level occupancy probability for each state

}

int.theta ~ dnorm(0, 0.1) # Priors: Intercepts for occupancy among subunits

int.p ~ dnorm(0, 0.1) # Priors: Intercepts for detection probability

beta.visit ~ dnorm(0, 0.1) # Priors for 1^st^_visit detection covariate

beta.day ~ dnorm(0, 0.1) # Priors for day of year detection covariate

beta.day2 ~ dnorm(0, 0.1) # Priors for day of year detection covariate (quadratic)

beta.dev_subunit ~ dnorm(0, 0.1) # Priors for area of developed land covariate at sub-unit level

beta.neigh ~ dnorm(0, 0.1) # Priors for number of occupied neighbors covariate at 10x10km grid cell level

beta.dev ~ dnorm(0, 0.1) # Priors for area of developed land covariate at 10x10km grid cell level

# 'Likelihood' (model structure)

for (i in 1:n.grid){

# Occurrence in grid i

z[i] ~ dbern(psi[i])

logit(psi[i]) <- int.psi[state[i]] + beta.neigh * neighbor[i] + beta.dev * developed_land[i]

for (j in 1:n.unit){

# Occurrence in sub-unit (sub-unit=unit=sample unit) j

a[i,j] ~ dbern(mu.a[i,j])

mu.a[i,j] <- z[i] * theta[i,j]

logit(theta[i,j]) <- int.theta + beta.dev_subunit * subunit_developed_land [i,j]

for (k in 1:n.survey){ #Surveys following first detection are coded as NA and excluded from likelihood

# Survey detection error process in sample k

y[i,j,k] ~ dbern(mu.y[i,j,k])

mu.y[i,j,k] <- a[i,j] * p[i,j,k]

logit(p[i,j,k]) <- int.p + beta.visit * first_visit[i,j,k] + beta.day * day[i,j,k] + beta.day^2^ * day[i,j,k]* day[i,j,k]

}

}

tmp[i] <- step(sum(a[i,])-0.1)

}

# Derived quantity

sum.z <- sum(z[]) # Estimated number of 10x10km occupied grids in sample

} # end model

Land Cover Quantification for Sampling Unit Selection

Land cover quantification of known rusty patched bumble bee (RPBB, *Bombus affinis*) locations. The US Fish and Wildlife Service (USFWS) manages a dataset of RPBB detections, consisting of 20,667 records from 1900 to present (Ellis et al. 2025). In April, 2022 we requested all RPBB records collected from 2017 to 2021. The USFWS database was created by extracting the RPBB records from the Bumble Bees of North America (Williams et al. 2014), supplemented by a combination of observations collected for a variety of purposes. The database includes RPBB observations submitted to Biodiversity Information Serving Our Nation (BISON) and by entities that hold scientific research permits under section 10(a)1(A) of the Endangered Species Act, state natural resource agencies (e.g., Wisconsin Bumble Bee Brigade), and Bumble Bee Atlas projects. The database also includes verified or science-grade incidental observations submitted to BumbleBeeWatch.org, iNaturalist.org, BeeSpotter.org, and directly reported to USFWS. We defined a 500-m radius buffer around each RBBB location in Illinois, Minnesota, and Wisconsin, for all points between 2007 and 2021. We then quantified land cover within each point buffer by tabulating the 2021 National Land Cover Database (NLCD). We calculated count of pixels by all classes within the buffer with the `rasterstats` package in Python and included only pixels with a centroid within each point buffer. We reclassified following land cover classes: 1) Developed Open Space, Developed Low Intensity, and Developed Medium Intensity to “Developed Low Intensity”, and 2) Hay/Pasture, Shrub/Scrub, Herbaceous and Herbaceous Wetland to “Grass/Shrub”. We then converted pixel counts to a percent total area within each buffer.

Results

The top three dominant land covers around RPBB points were Low Intensity Developed Land, Grass/Shrub and Deciduous Forest (Figure S1). We focused our initial selection of subunits for 2022-2024 sampling on areas containing Low Intensity Developed Land and Grass/Shrub. We did not consider selecting areas with Deciduous Forest because of the difficulties with finding summer worker bumble bees in forests.


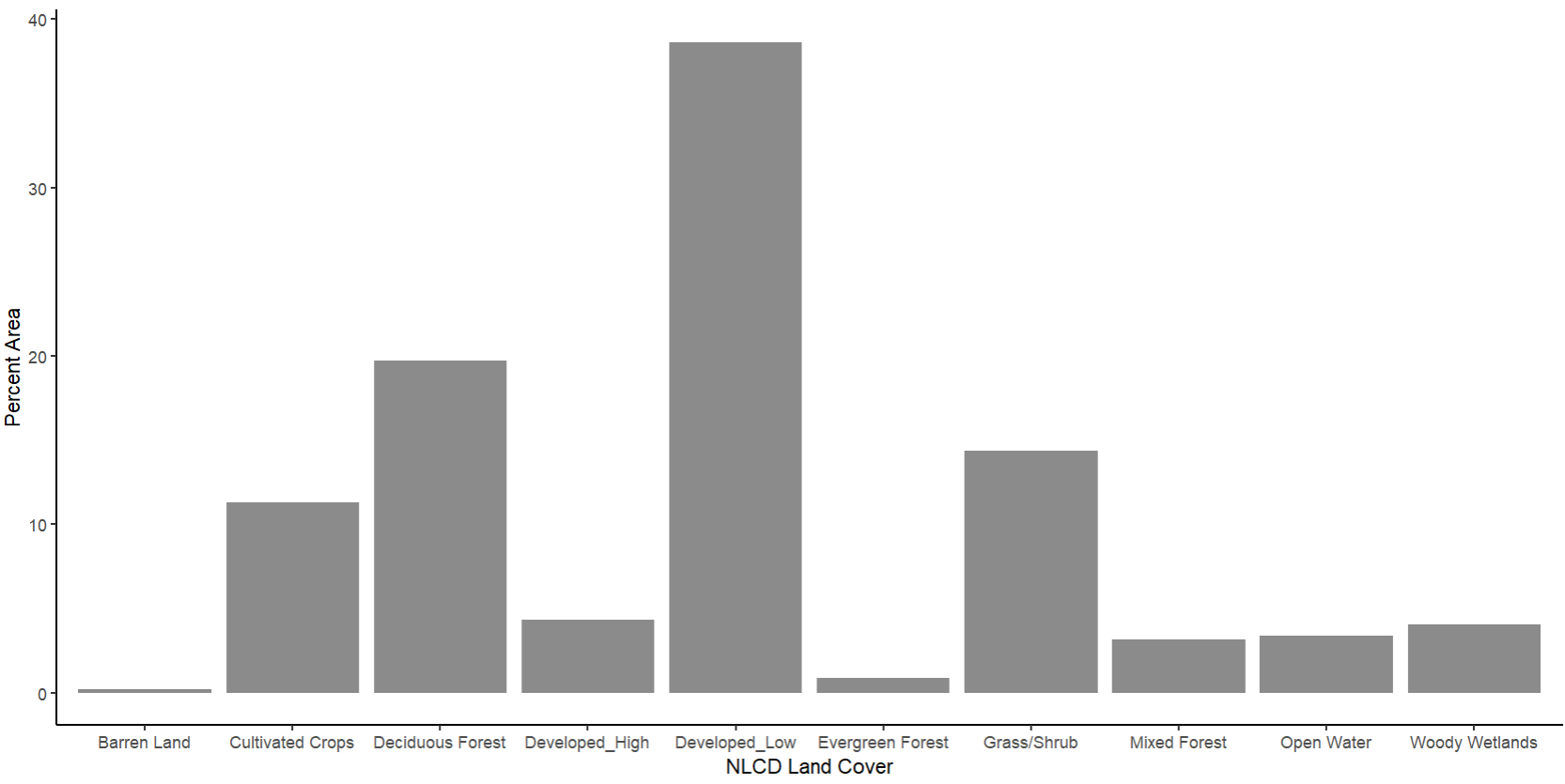


Figure S1. Land cover quantification of all known *Bombus affinis* presence points in Minnesota, Illinois and Wisconsin from 2007-2021. The USGS National Land Cover Database was used to quantify land cover within 500m of all recorded points. RPBB occurrence records provided by the US Fish and Wildlife Service.

A


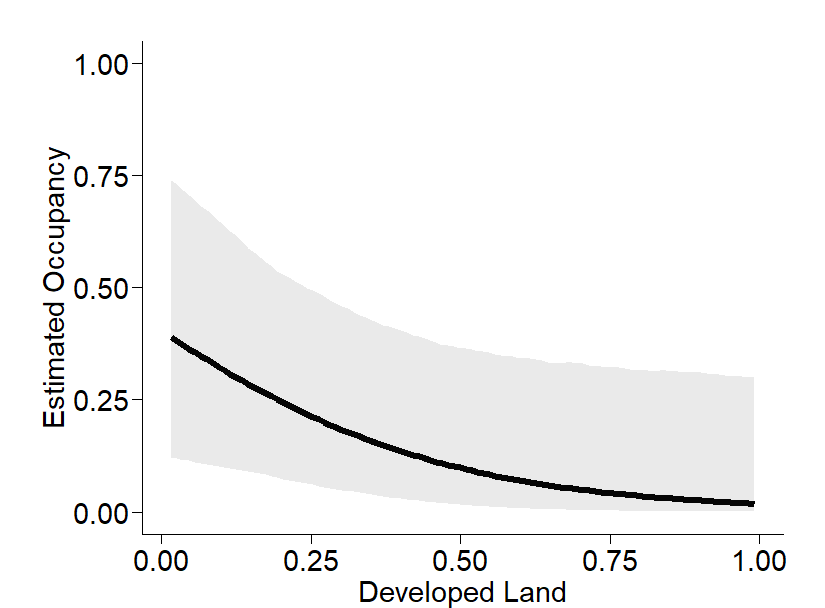


B


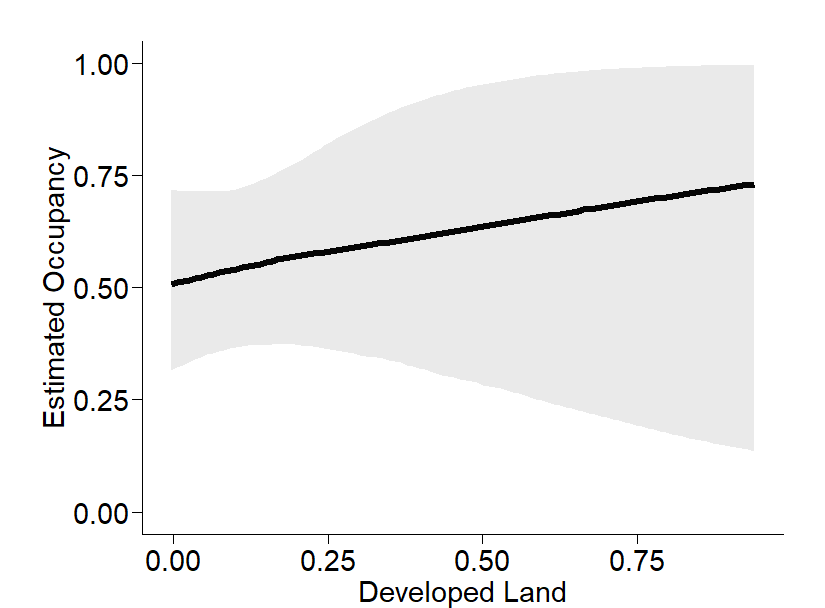


Fig. S2. Relationship between the area of developed land and rusty patched bumble bee (*Bombus affinis*) occupancy at A) 100km^2^ grid cells and B) 3.14ha circular or 150m roadside transect, subunits. Shaded gray region represents the 95% credible intervals (CI). Parameter estimates for the *developed land* covariate were -0.86 (-1.86 ˗ 0.03, 95% CI) and 0.31 (-0.59 ˗ 1.56) for grid cell and subunit scales, respectively.

A


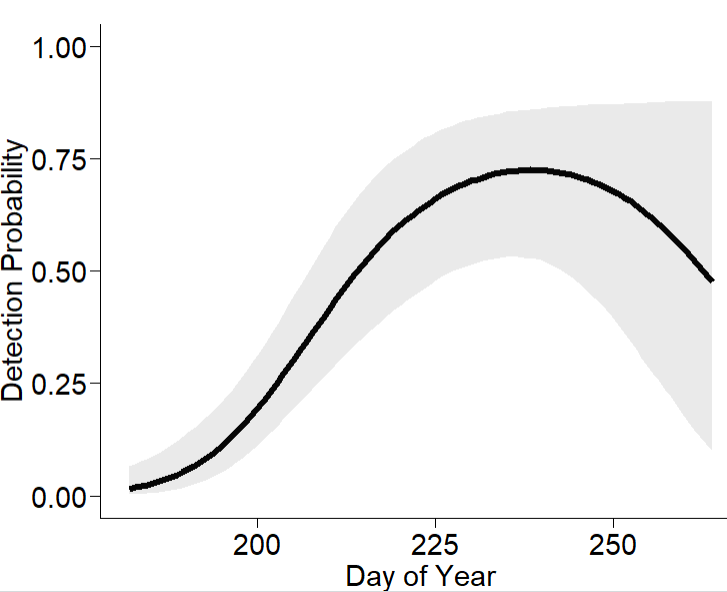


B


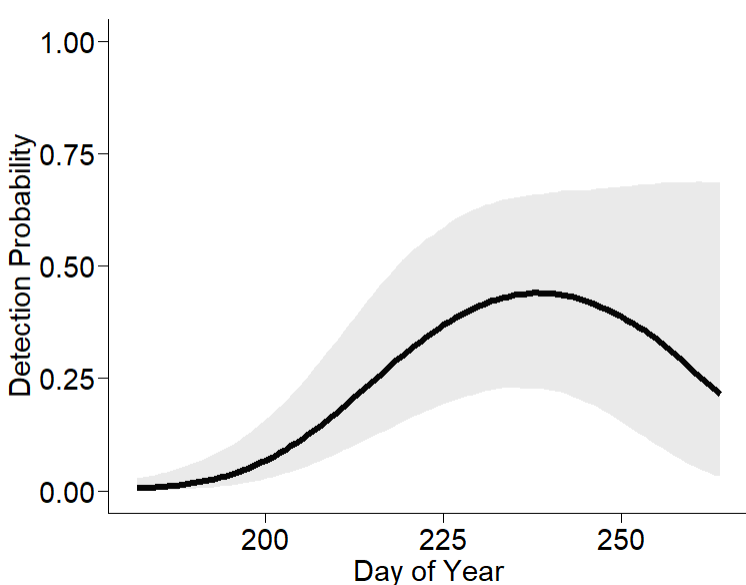


Fig. S3. Relationship between the day of year and rusty patched bumble bee (*Bombus affinis*) detection during a 30-minute visual encounter survey conducted during the first visit (A) or second or third visit (B) to a subunit. Shaded gray region represents the 95% credible intervals.
